# Clinical Applications, Legal Considerations and Implementation Challenges of Smartphone-Based Thermography: A Scoping Review

**DOI:** 10.3390/jcm13237117

**Published:** 2024-11-25

**Authors:** Alessandra Putrino, Michele Cassetta, Mario Raso, Federica Altieri, Davide Brilli, Martina Mezio, Francesco Circosta, Simona Zaami, Enrico Marinelli

**Affiliations:** 1 Department of Oral and Maxillo-Facial Sciences, Sapienza University of Rome, 00161 Rome, Italy; alessandra.putrino@uniroma1.it (A.P.); michele.cassetta@uniroma1.it (M.C.); federica.altieri@aslroma1.it (F.A.); brilli.1846837@studenti.uniroma1.it (D.B.); martina.mezio@uniroma1.it (M.M.); 2 Department of Computer Science, Sapienza University of Rome, 00161 Rome, Italy; mario.raso@uniroma1.it; 3 Dental Unit, ASL Roma 1, 00161 Rome, Italy; 4 Department of Clinical, Internal, Anesthesiological and Cardiovascular Sciences, Sapienza University of Rome, 00161 Rome, Italy; francesco.circosta@uniroma1.it; 5 Department of Anatomical, Histological, Forensic and Orthopedic Sciences, Sapienza University of Rome, 00161 Rome, Italy; 6 Department of Medico-Surgical Sciences and Biotechnologies, Sapienza University of Rome, 04100 Latina, Italy; enrico.marinelli@uniroma1.it

**Keywords:** thermography, body temperature, diagnostic imaging, medical devices, diagnosis, smartphone, healthcare, health evaluation, legal issues, litigation

## Abstract

Medical thermography is a non-invasive technique that allows the measurement of the temperature of the human body surface, exploiting the heat emitted by the body through the skin in the form of infrared electromagnetic radiation. Recently, smartphone-based thermography (ST) has drawn considerable attention. This scoping review (SR) aims to describe its current applications and reliability based on currently available research findings, also taking into account the medico-legal implications linked to its use. A search of the sources was conducted on multiple databases (PubMed, Scopus, Cochrane, Lilacs, Google Scholar). Based on a set of eligibility criteria, all articles deemed useful were included in the SR. Collected data, processed with descriptive statistics, are then discussed. From the initial 241 results, after duplicate removal and full-text reading based on inclusion/exclusion criteria, 20 articles were classified according to the main characteristics and indications and outcomes are highlighted based on clinical evidence. The most frequently documented fields of ST are wound care management and vascular surgery. Other disciplines are less explored (dentistry, ophthalmology, otorhinolaryngology, orthopedics, etc.). Practicality, operational simplicity and affordability of mobile thermographic devices are the chief strengths of this technology. Comparative studies with traditional thermal imaging methods are poor in terms of the number of patients analyzed but this technology showed high sensitivity and accuracy in the large number of patients enrolled in observational studies, encouraging the development of further operational protocols in all medical specialties. Gaining a deeper understanding of such techniques will also help settle the medico-legal issues which may arise from the clinical implementation of ST, thus appraising its reliability and safety from that perspective as well.

## 1. Introduction

In nature, thermal energy is transmitted in an interconnected way by conduction, convection and radiation [1]. Heat transmission by radiation stems from thermal radiation from an object, including the human body, to another or to its surroundings [2]. Heat transfer by radiation is highly relevant for thermography. Any thermographic investigation is in fact possible only thanks to this basic heat transmission principle [3]. Thermography applications in medicine are based on the correlation between the functional body conditions and its internal temperature [3,4]. Normally, the body can maintain its temperature at a constant level, even if the external thermal conditions vary. Excess heat is dispersed outside mainly through the skin. The skin releases thermal energy into the surrounding environment as does any other object with a temperature over zero [5]. 

Thermal imaging cameras detect these temperatures and create images from which surface body temperatures can be identified. The diagnostic value of such a tool in some conditions [6] stems from its quickness, non-invasivity and reliability to identify real-time “hot spots”: visual maps of the thermal gradients existing on the body’s surface [7]. Tissue metabolism and blood circulation play a key role in regulating body temperature, which is also affected by the nervous and endocrine systems. Pathological or physiological alterations can be detected and visualized by thermographic images in which blood flow fluctuations, with varying intensity levels, can be highlighted [8]. In human medicine, thermography already constitutes an adjunctive diagnostic avenue for early and non-invasive diagnosis in angiology, cardiology, dentistry, gastroenterology, nephrology, oncology, respiratory and infectious diseases [9,10,11,12,13,14,15,16,17]. In recent years, smartphone use has increasingly supported medical education and interventions [18,19,20,21]. Smartphone-based thermal cameras for medical purposes have been introduced as a cost-effective alternative to more expensive and cumbersome thermographic instruments [22,23]. Legal issues may, however, arise from medical thermal scanning, especially if misdiagnoses should occur [24,25,26]. This scoping review aims to identify current medical applications of smartphone-based thermography in order to assess the reliability of this mobile technology based on currently available research findings and medico-legal implications related to its use.

## 2. Materials and Methods

**Figure 1:**
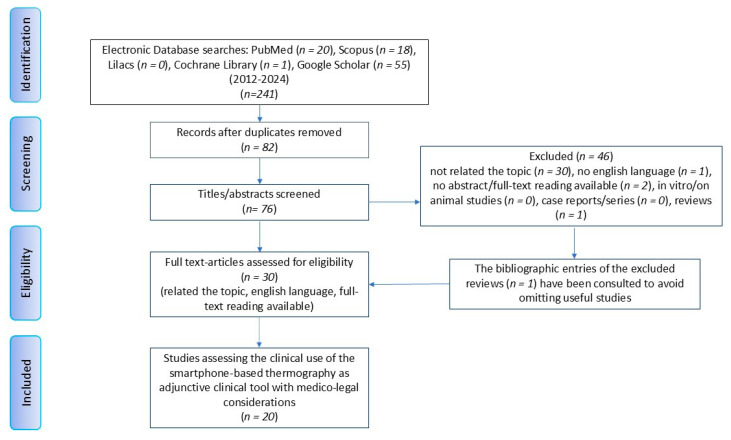
Review process in compliance with PRISMA-ScR guidelines.

This scoping review project was registered as an open-ended registration on OSF Registries (registration DOI: 10.17605/OSF.IO/R36D8) and was conducted following the PRISMA-ScR guidelines [27]. The vetting and selection of sources lasted from 28 February 2024 to 8 November 2024. Five relevant databases were drawn upon: PubMed, Scopus, Cochrane Library, Google Scholar and Lilacs (Figure 1). The research query was set following the population/problem, concept, context (PCC) approach. This scoping review aimed to determine whether patients under different specialist care may benefit from a reliable and safe, including from a medico-legal standpoint, complementary diagnostic examination through the use of smartphone-based thermography (Table 1). The search was conducted using the following MeSH terms, search strings and free terms in combination with the Boolean operators “AND” and “OR”: thermography, body temperature, diagnostic imaging, diagnosis, medical devices, smartphone, cell phone, medico-legal, forensic and litigation. The sources were analyzed by three expert independent operators in different fields of medicine (A.P., F.C. and S.Z.), applying the established eligibility criteria. The studies ultimately selected for the purpose of this review article were randomized and non-randomized comparative clinical studies, observational studies, prospective studies and proof-of-concept studies. In vitro and in vivo (on animals) studies, case reports, reviews, editorials and commentaries were not eligible for inclusion (Table 2). Only open-access studies in English were considered. To avoid leaving out suitable studies, the sources cited by each selected study were also vetted. No restrictions on publication year were set. Any duplicates were merged and eliminated by Zotero Software (Zotero 5.0 for Windows, Corporation for Digital Scholarship, Vienna, VA, USA). Research articles with dubious contents in the abstract were included for full-text reading and eventually assessed by the reviewers independently. Any disagreement on a given article was discussed and solved by the reviewers with the contribution of three other expert clinicians (M.C., F.A. and E.M.). A computer science specialist (M.R.) contributed to shedding light on technical contents. Final data were firstly independently framed and then discussed and merged as unique results. No quality assessment tools were used to evaluate the review sources since this is not required for this type of review. Data from each study were summarized by study design, population samples, medical branch of application, intervention and outcome.

## 3. Results

For this scoping review, 241 studies of potential interest were identified, published between 2012 and 2024. After weeding out duplicates from the different databases (*n* = 82), 76 articles were screened in detail, 30 of which met the eligibility criteria. Following this process, only 20 studies published between 2016 and 2024 were included in the scoping review. The flow chart diagram (Figure 1) outlines the search strategy by which this review was framed. Several fields of interest emerged, most of which were multidisciplinary: microsurgery [28], wound care management [29,30,31,32,33,34], dentistry [35], ophthalmology [36], psychophysiology [37,38], otorhinolaryngology [39,40], vascular surgery [41,42,43], plastic surgery [22,44], orthopedics [45] and neurosurgery [46]. As for the study design, the research articles included in this review were defined by their authors as prospective studies [28,32], comparative studies [30,37], cross-sectional studies [29,36], proof-of-concept studies [22,31,40], observational studies [33,35,38,39,41,42,43,44,46] and a randomized controlled trial [34]. The total number of subjects totaled 647 [29,30,31,32,33,34,35,36,37,39,40,41,42,43,44,45,46], though two studies did not provide this information [22,38]. Smartphone-based thermography only involved adult patients; gender was not always specified. All our sources provided only incomplete information about their protocols and environmental temperature calibration prior to preoperative, intraoperative and postoperative recordings. In each field of application, with the exception of one study [42], all outcomes showed thermography via smartphone-based mobile devices as a substantially useful and reliable tool for adjunctive diagnostic and follow-up examination (Table 3). However, in their related discussions, the studies did mention possible medico-legal issues arising from such applications.

## 4. Discussion

Thermography is a non-invasive test mostly performed for diagnostic purposes. Its use may also improve peri- and postoperative clinical assessments. Recent advances in mobile technologies led to the development of professional use of smartphones to support clinical activity in general as well as in some specific medical branches [12]. All the research articles included herein had several common limitations. In fact, all but three [34,37,41] exhibited a lacking methodological approach that highlights the accuracy of smartphone-based thermography as opposed to conventional thermography or comparable examinations. Such a flaw prevents a critical evaluation of the technical performances of such devices. This limitation should not be discounted, in addition to the rather small sample size of most studies and the fact that none of them provided the determination of the sampling power. Hence, the clinical value and generalizability of the results thus obtained are rather limited. Almost all studies used the same devices with a ±3 °C accuracy level and 150 mK thermal sensitivity, allowing for the detection of temperature differences as small as 0.15 °C. However, from an experimental point of view, apart from one study [35], no studies mentioned the need for environmental calibration of the device. It is therefore reasonable to assume that the studies did not account for important surrounding factors capable of significantly swaying the results (i.e., environmental temperature, acclimatization and calibration based on the emissivity of tissues and biological liquids). Furthermore, aspects such as the device’s technical features and operative system (Android or iOS), which could affect thermal devices, were never addressed. A major issue with this has to do with the reliability of the apps/programs available on virtual stores, the protection of third-party data (i.e., patient data) that are possibly acquired and the risk of non-expert operators, including the patients themselves, misusing medical resources which can result in misdiagnoses based on an inaccurate “do-it-yourself” approach [18,19,47]. 

Smartphone-based thermography in medicine has spread significantly in recent years, mostly due to its accessibility, affordability and non-invasive nature [29,37]. Numerous studies have explored its potential across different medical applications that could transform both diagnostic and therapeutic practices [22,28,32,39]. Several medical specialties could benefit from this affordable and non-invasive method, including gynecology and pediatrics, e.g., the evaluation of post cesarean section recovery in obese women [48] or body temperature measurement for children undergoing intensive care or with rheumatic conditions [49,50]. As our findings show, only one study considered the pediatric population as target, for febrile measurements [35], pointing to a considerable potential for further growth.

Previous studies have also investigated the medico-legal reliability of traditional thermography for diagnostic purposes [24,25,26,27], which is still quite controversial for smartphone-based thermography. The integration of smartphone-based thermal imaging technology into clinical practice has gained notable traction over the past decade, reflecting a shift towards more accessible and cost-effective diagnostic tools [23,33,34,35,43,46]. Various studies have acknowledged its value in a host of medical branches, emphasizing its role in detecting clinical conditions and monitoring postsurgical outcomes [22,23,24,25,26,27,28,29,30,31,32,33,34,35,36,37,38,39,40,41,42,43,44,45,46]. Hardwicke et al. [22] and Ko and Chiu [23] demonstrated the potential of smartphone thermography in identifying perforators, suggesting that this technology could enhance surgical precision and improve flap viability in reconstructive surgeries. Such findings were corroborated by subsequent research indicating the effectiveness of smartphone thermography in assessing lower extremity ischemia, as outlined by Lin and Saines [41]. This seems to point to smartphone-based thermal imaging as a valuable vascular assessment option. 

Additionally, several innovative uses of smartphone thermography, beyond traditional applications, have been researched as well. Kanazawa et al. [28] explored its role in detecting subclinical inflammation, while Ban et al. [29] applied thermal imaging to identify peritonsillar abscesses. These studies indicate that smartphone thermography could facilitate early diagnosis, thereby potentially reducing complications associated with delayed treatments. Chen et al. [32] further illustrated the evolution of this technology, and Cho et al. [33] highlighted the comparative effectiveness of smartphone-compatible thermal imaging with traditional imaging modalities, such as computed tomography angiography. This source adds to the growing body of evidence pointing to smartphone thermography as a reliable adjunct in clinical assessments, particularly in trauma and emergency settings. The adaptability of smartphone thermal imaging extends to mental health as well. Cho et al. [37] utilized this technology to assess perceived mental stress, highlighting its remarkable versatility in a multidimensional approach to patient wellness. Similarly, studies by Minatel Riguetto et al. [34] and van Doremalen et al. [35] have presented compelling evidence supporting smartphone thermography in routine clinical practice, e.g., for the monitoring of chronic conditions. Moreover, a recent investigation into the use of smartphone thermography during the COVID-19 pandemic by Putrino et al. [40] has shed light on its capacity to adapt swiftly to emerging health crises. Such a degree of adaptability speaks volumes as to this technology’s potential as a standard tool for healthcare monitoring in various environments. In terms of future prospects, mobile smartphone-based thermography could take on an increasingly significant role as a diagnostic monitoring tool in telemedicine, thanks to its cost-effectiveness and ease of use [51]. As the body of evidence supporting smartphone thermography continues to grow, further large-scale studies and randomized controlled trials, such as those conducted by Qin et al. [45] and Harhangi et al. [46], will be needed to lay the groundwork for standardized implementation protocols. The promising results from these trials suggest that as smartphone technology continues to evolve, it will likely play an increasingly crucial diagnostic and patient-monitoring role across various medical specialties. The approach proposed by Harhangi et al. [46] provides a non-invasive, quick and cost-effective method for monitoring shunt function, potentially reducing the need for more invasive and expensive imaging techniques such as CT scans. Given the importance of timely shunt intervention, this application could improve patient outcomes by enabling earlier detection of complications. As for surgical recovery, Li et al. [44] explored the use of smartphone-based infrared thermography to monitor the healing of thoracic surgical incisions. Their preliminary findings point to thermography as effective at identifying thermal changes linked to inflammation and infection. Consequently, this technology offers a valuable tool for postoperative care, particularly in remote or resource-limited settings where frequent in-person consultations may not be feasible. 

Moreover, remote monitoring of wound healing could enhance patient safety and reduce the burden on healthcare systems. Qin et al. [43] focused on the preventive aspect of healthcare by examining the effectiveness of a thermography-driven protocol in reducing the recurrence of diabetic foot ulcers. Their study, which was conducted in underresourced settings, found that regular thermographic monitoring could identify early signs of foot ulcers, allowing for timely interventions and reducing the risk of severe complications. This application is particularly relevant in managing chronic conditions like diabetes, where early detection and prevention can greatly improve outcomes and reduce healthcare costs.

No comprehensive literature reviews on smartphone-based thermography in medicine are currently available, with the exception of one by Van Dieren et al. [52], i.e., a systematic review and meta-analysis centered on smartphone thermography in flap surgery, specifically for the identification of perforators. Their analysis confirmed that smartphone-based thermography could be a reliable method for preoperative planning, particularly in identifying suitable perforators for flap surgeries. This capability appears to be crucial for the success of such procedures, as accurate perforator identification lowers the risk of flap failure and associated complications. These findings suggest that thermography could enhance surgical precision, leading to better patient outcomes. 

Collectively, these studies illustrate the broad applicability and potential benefits of smartphone-based thermography in medicine. Whether in diagnostic assessments, surgical planning, postoperative monitoring or preventive care, smartphone thermography may offer a versatile tool likely to enhance patient care across a range of settings. However, while the initial findings are promising, further research is necessary to establish standardized protocols, validate these techniques in larger and more diverse populations and address potential limitations, such as the influence of external factors on thermal readings. This is also significant when taking into account medico-legal issues related to diagnostic errors and the information about its value, as any adjunctive diagnostic tool needs to be understood by patients and clarified when providing informed consent [53]. Image quality may be an issue: despite generally good resolution levels, images from thermal cameras can turn out unclear, especially due to the surrounding environment. This may detract from the clinical value of the data thus collected [54]. Many methods have been proposed, but one of the most recent uses, the “convolutional neural network,” is a denoising method that can retain remarkable levels of infrared image detail [55,56].

As technology continues to evolve, smartphone-based thermography can potentially become an integral part of modern medical practice, a powerful tool for improving patient outcomes and expanding high-quality care access. As described above, the limitations of this study mostly have to do with the limited number of sources and the restricted medical fields of application, both of which detract from the study’s ability to conclusively identify this technology as a reliable method for each medical specialty. 

The medico-legal relevance of such innovations, which are currently unregulated by national or international healthcare systems and frameworks or by accredited scientific societies, seems to be rarely mentioned by the studies herein accounted for [22,29,30,31,32,33,34,35,36,37,38,39,40,41,42,43,44,45,46]. While such sources were rather limited and questionable from a methodological standpoint, at least some of them addressed this topic and pointed to the need to handle recent technologies with caution when it comes to acknowledging their diagnostic potential. Before corroborating their validity in terms of accuracy and reproducibility of the results and their interpretation, as in any other diagnostic imaging test, the medico-legal implications may be underestimated. Relying on data extracted from thermographic images from smartphone devices, on whose postacquisition management the sources appear to be quite ambivalent, could significantly influence clinical evaluation; this in turn may contribute to clinical, diagnostic and prognostic errors that inevitably damage patients and expose medical professionals to negligence-based malpractice claims from the use of unproven or misleading technologies. There is some precedent on record regarding traditional thermography, which is especially meaningful because such technologies can profoundly impact healthcare systems [57,58]. Furthermore, recent experiments aimed at evaluating the diagnostic power and accuracy of cameras relying on deep network technologies and convolutional neural networks could be helpful in estimating and improving the reliability of smartphone-based thermography [59,60].

## 5. Conclusions

In conclusion, research findings seem to support smartphone-based thermography as a valuable asset in modern medical practice. As its application spreads, ongoing research and development will be essential to maximize its potential and integrate it effectively into routine clinical frameworks. To more fully understand this technology and its implications, it is advisable to further substantiate the results presented herein by encouraging comparative studies with similar technologies, such as traditional thermography, and with randomized clinical studies with larger and more diverse samples, both in terms of patient age and application scope. This can be more easily achieved thanks to the very nature of such approaches: they are not invasive and do not interfere with standard diagnostic and operational procedures. Instead, they can make for an additional test which must be considered as such, including from a medico-legal perspective. Only through broader experience can this thermographic approach be brought to the attention of healthcare policymakers and regulatory bodies and, over time, validated and recognized for diagnostic purposes. To that end, it will be essential to identify specific applications, usage protocols and accuracy standards before such devices can become mainstream for professional medical use, even in the ever-evolving, highly innovative field of telemedicine.

## Figures and Tables

**Table 1:** Research formulation based on the Population/Problem, Concept, Context (PCC) strategy.

Population/Problem	Patients Under Specialist Care
Concept	Smartphone-based thermography is reliable and safe from a medico-legal standpoint as well
Context	Complementary tests in diagnostic path

**Table 2:** Eligibility criteria.

Inclusion Criteria	Exclusion Criteria
Randomized and non-randomized comparative clinical trials	In vitro and in vivo (animal studies), case reports/case series, reviews, editorials, commentaries
English language	Other languages
Abstract and full text available	No abstract and/or full text available

**Table 3:** Review sources listed by year of publication.

Authors, Year, Country	Field of Interest	Context	Study Design	Sample Size	Intervention	Outcome
Hardwicke et al., 2016, UK [22]	Plastic surgery	Detection of perforators	Proof-of-concept study	n.s. patients and volunteers	Pre-, intra- and postoperative thermograms can assist in planning, executing and monitoring free flaps	Smartphone thermography is a promising adjunctive tool in diagnosis and operative surgery
Kanazawa et al., 2016, Japan [28]	Wound care management	Assessment of inflammation based on temperature increase compared with thermography used in pressure ulcer (PU) and diabetic foot	Pilot cross-sectional observational study	8 patients with PU and diabetic foot	Comparison of thermal images between smartphone device and hand-held device	Thermography can work as alternative technique at the patients’ bedside
Ban et al., 2017, Korea [29]	Otorhinolaryngology	Detection of peritonsillar abscesses	Observational study	6 patients	Open thermal photographic images were taken preoperatively	Hot spots with higher temperature in peritonsillar areas were identified as abscesses
Lin et al., 2017, USA [30]	Vascular surgery	Assessment of lower extremity ischemia	Observational study	8 patients with lower extremity arterial occlusive disease	Thermal photographic images were taken pre-, intra- and postoperatively of endovascular intervention or surgical bypass procedure and compared to ultrasound assessment	This smartphone-based camera device is non-invasive, easy to use and cost-effective in assessing patients with lower extremity tissue perfusion
Xue et al., 2018, USA [31]	Wound care management	FLIR ONE’s reliability in burn wound assessment	Comparative study	5 patients with acute third-degree burn wounds	This case series investigates the accuracy of FLIR ONE in comparison with the widely used indocyanine green (ICG) angiography in assessing burn wounds before surgical intervention	There is a strong correlation between smartphone-based thermography and ICG when assessing salvageable tissue in third-degree burn wounds. FLIR ONE maximizes the convenience and cost-effectiveness of infrared thermography technology but may overestimate unsalvageable tissue area. Mobile thermography is promising as an adjunct to current imaging modalities
Chen et al., 2019, China [32]	Vascular surgery/oral and maxillofacial surgery	To investigate the value of smartphone-based thermography in mapping peroneal artery perforators	Observational study	12 patients enrolled for fibular flap	Lower limbs were first studied using smartphone-based thermography, then hot spots were marked with rubber bands and CT scan was performed	Sensitivity and predictive value of smartphone-based thermography are low. It can be used as adjunctive tool
Cho et al., 2019, UK [33]	Psychophysiology	Stress detection by thermal imaging	Comparative study	17 participants	Blood volume pulse and vasoconstriction/dilatation-induced temperature changes were measured after stress-inducing mental workload tasks (after 20 seconds) with smartphone-based thermography, comparing results with their perceived stress levels using a 10 cm visual analogue scale	The results demonstrate the feasibility of using smartphone-based imaging for instant stress detection
Riguetto et al., 2019, Brazil [34]	Ophthalmopathy	Ocular temperature assessment for measuring inflammatory activity in Graves’ ophthalmopathy and its correlation with clinical activity score (CAS)	Cross-sectional study	136 Graves’ disease patients and 62 healthy controls	Exophthalmometry, CAS and thermal images from caruncles and upper eyelids were acquired from all subjects	IRT was an objective and simple tool for evaluation and follow-up of inflammation in GO. Patients with significant inflammatory activity were evidenced. Good correlation with the CAS was found in 12 months of observation
van Doremalen et al., 2020, the Netherlands [35]	Wound care management	Thermal imaging for inflammation detection in diabetic foot disease	Proof-of-concept study	8 patients with diabetic foot ulcer	Three-dimensional (3D) models with thermal infrared images obtained with three smartphone-based thermal infrared cameras were aligned using a high-resolution medical 3D imaging system to map thermal images onto the 3D model to create the 3D visualizations and to assess their quality and validity	Future developments are expected to improve the image-processing techniques, leading to easier-to-use hand-held applications and driving further research
Alisi et al., 2021, Jordan [36]	Orthopedics	Detection of lower limb reperfusion post total knee arthroplasty (TKA)	Prospective study	46 patient undergoing primary TKA	A thermographic camera captured images at ankle joint preoperatively and at 1, 10 and 20 minutes post tourniquet release on operation side. The contralateral ankle was a control	Infrared thermography via a smartphone-connected camera is a simple, non-invasive, feasible and reliable technology. It can provide an objective measure
Baran, 2021, Poland [37]	Psychophysiology	Stress detection by thermal imaging	Observational pilot study	Not specified	Performing thermographic analysis during a stressful situation watching a movie	Stress photography is a promising method of monitoring human stress
Pereira et al., 2021, USA [38]	Microsurgery	Flap perforator mapping	Prospective study	25 patients	Smartphone thermography was used in all patients preoperatively to identify ideal perforator or vascular network "hot spots" that allowed appropriate flap design. Intraoperative and postoperative monitoring was also performed	Smartphone thermography is an inexpensive and expeditious means for the identification of "hot spots" and to ensure perfusion to lower extremity perforator local flaps. It is a complementary technique for their safer design, harvest and subsequent monitoring in conjunction with more complex screening tools as indicated
Phillips et al., 2021, USA [39]	Vascular surgery/plastic surgery	To assess smartphone-based thermography in detecting microvascular flow insufficiencies	Observational study	19 patients needing deep inferior epigastric artery perforator (DIEAP) free flaps	Images were obtained pre- and intraoperatively and at instances of concern for flap viability	Smartphone-based thermography is useful to recognize microanastomotic failure and free flap perfusion
Putrino et al., 2021, Italy [40]	Dentistry/public health	To assess smartphone-based thermography in identifying body temperature alterations during SARS-CoV-2 pandemic	Observational study	30 orthodontic patients undergoing clinical check	Forehead digital thermometer temperatures and smartphone thermal camera images were taken of ear areas and inner canthi	A thermal camera on a smartphone is a reliable tool for measuring body temperature and its use is interesting in pediatric dentistry. Mobile thermographic values of ears and inner canthi areas can be used as an alternative to forehead digital thermometer measurements
Shokri et al., 2021, USA [41]	Otorhinolaringology/plastic surgery	Perfusion dynamics in pedicled and free tissue reconstruction assessed by smartphone-based thermography and laser fluorescence video angiography (FA)	Proof-of-concept study	4 patients	Tissue perfusion was assessed intraoperatively with thermography and FA	Smartphone-based thermography is useful in early detection of poor flap viability
Zenunaj et al., 2021, Italy [42]	Vascular surgery	To evaluate foot temperature changes in atherosclerotic peripheral arterial disease (PAD) before and after revascularization	Observational study	40 patients	Thermographic measurements on the foot (anterior tibial, podal, posterior, arcuate arterias) with smartphone and duplex scan with ankle brachial index (ABI) calculation	Smartphone-based IRT is reliable to assess foot blood perfusion in symptomatic PAD patients and during the follow-up after revascularization
Qin et al., 2022, Japan [43]	Wound care management	Plantar thermal pattern in diabetic foot ulcer risk assessment	Prospective study	10 healthy young volunteers	Plantar thermal images using a smartphone attached to a selfie stick were taken at different times of day for 4 days in home settings	The medial arch pattern was the most common hot area, which matches previous findings in well-controlled clinical settings. Smartphone-based thermography may be feasible as a self-assessment tool in the home setting
Li et al., 2023, China [44]	Wound care management/thoracic surgery	Early assessment of healing progress and potential for predicting the healing status of thoracic surgical incisions	Observational study	40 patients	Thermal image acquisition and temperature extraction were performed for 7 consecutive days postoperatively, and visualized early warning information was recorded	The rates of sensitivity (91.67%) and specificity (85.71%) indicate a promising clinical application of mobile thermography for assessing incision healing dynamics and providing a scientific basis for later artificial intelligence-driven decision algorithms
Qin et al.,2023, Japan [45]	Wound care management	Investigation of the efficacy of thermographic evaluation with a smartphone in preventing diabetic foot ulcer recurrence	Randomized controlled trial	120 patients	Thermography evaluation was performed to assess baseline risk. Then, personalized foot care and education were conducted monthly in patients with increased foot lesion baseline temperature	Foot care and personalized education delivered at a frequency based on the risk level from the assessment of foot lesions and personalized care and education supported by thermography with a smartphone improve quality of life and care and induce a reduction in diabetic foot ulcer recurrence
Harhangi et al. 2024, the Netherlands [46]	Neurosurgery	Detection of shunt patency in patients with hydrocephalus	Observational study	51 patients with a shunt for hydrocephalus without suspected dysfunction	The thermographic camera clearly detected the flow of cerebrospinal fluid in the cooled shunt trajectory	Smartphone-based video thermography (FLIR ONE video camera) may be a simple alternative to show shunt patency without exposure to radiation
